# Family history of breast cancer as a second primary malignancy in relatives: a nationwide cohort study

**DOI:** 10.1186/s12885-021-08925-y

**Published:** 2021-11-12

**Authors:** Guoqiao Zheng, Jan Sundquist, Kristina Sundquist, Jianguang Ji

**Affiliations:** 1 Center for Primary Health Care Research, Lund University/Region Skåne, Jan Waldenströms gata 35, 205 02 Malmö, Sweden; 2 Department of Family Medicine and Community Health, Department of Population Health Science and Policy, Icahn School of Medicine at Mount Sinai, New York, USA; 3 Center for Community-based Healthcare Research and Education (CoHRE), Department of Functional Pathology, School of Medicine, Shimane University, Izumo, Japan

**Keywords:** Breast carcinoma, Cancer incidence, Familial clustering, Multiple primary cancer

## Abstract

**Background:**

With the increasing number of breast cancer (BC) diagnosed as a second primary malignancy after a first primary non-breast cancer (BCa-2), it is unclear about the familial risk of BC among women with a first-degree relative (FDR, parents or siblings) affected by a BCa-2.

**Methods:**

In this Swedish nationwide cohort study, 5315 women with a FDR affected by BCa-2 and 115,048 women with a FDR affected by BC as the first primary cancer (BCa-1) were followed for the first primary invasive BC diagnosis. Relative risk (RR) of BC was estimated through Poisson regression by using 2,743,777 women without a family history of cancer as reference. The risk was stratified by the diagnostic age of BC in FDR, proband type, the time interval between the first primary cancer and BCa-2 in FDR as well as the site of first primary cancer diagnosed in FDR before BCa-2. We also calculated the cumulative incidence of BC from birth to a specific age for the three groups.

**Results:**

The cumulative incidence from birth to age 70 was 10% among women with a family history of BCa-2. The RR of BC with a family history of BCa-2 (RR, 1.68, 95%CI, 1.49 to 1.88) was comparable to that with BCa-1 (1.68, 1.63 to 1.73). The risk was largely consistent irrespective of proband type. The age of onset of BCa-2 in FDR (RR _early-onset_, 1.72 vs. RR _late-onset_ 1.67) had less influence on the risk compared to BCa-1 in FDR (1.89 vs. 1.63). In the analysis stratified by the time between the first primary cancer and BCa-2 in relatives, the risks were largely similar. For the site of first primary cancer diagnosed in FDR before BCa-2, the increased BC risk was found in women whose FDRs were diagnosed with first primary gastric, colorectal, endometrial, ovarian, nervous system and endocrine gland cancers, and non-Hodgkin lymphoma.

**Conclusions:**

Women with a family history of BCa-2 have a similar overall BC risk as those with a family history of BCa-1. The risk varied according to the site of first primary cancer diagnosed in FDR before BCa-2.

**Supplementary Information:**

The online version contains supplementary material available at 10.1186/s12885-021-08925-y.

## Introduction

Breast cancer (BC) is the leading cause of cancer death among women worldwide [1]. Family history of BC in first-degree relative (FDR) is one of the important risk factors. The relative risk (RR) of BC in women was estimated at about 1.8 with one FDR affected by BC [2–4]. Many medical organizations recommend screening with mammography for women aged 40 or above with an average risk of BC. Most screening guidelines acknowledge the need for earlier screening for those at a higher risk [5], and the risk-adapted starting age has been proposed in patients with a family history of BC [6]. Unfortunately, no previous studies have considered the order of the primary BC. Multiple primary BC in a relative is a well-established risk factor. While there is limited evidence on the familial risk associated with the diagnosis of BC as a second primary malignancy after a cancer other than BC (BCa-2). The etiology of BCa-2 is not totally the same as BC diagnosed as a first primary malignancy (BCa-1). Some of the risk factors that lead to a diagnosis of BCa-2, such as treatment from the first primary cancer, are absent for BCa-1 and are not shared among family members. Despite that BC is the most common second primary cancer among women [7], it is unknown whether the familial risk remains unchanged when the FDRs are diagnosed with a BCa-2.

The aim of the study was to estimate the familial risk of BC among women whose FDR was diagnosed with BCa-2 after a first primary non-breast cancer. The findings may bridge the knowledge gap of the family history of BCa-2, given the increasing number of cancer patients diagnosed with BCa-2. It will also be important for the realization of personalized cancer prevention and early detection.

## Methods

### Data resources

This study was conducted with linkage of several Swedish national registers using each person’s unique identification number. To preserve confidentiality, this ID number was replaced by a serial number. The Swedish Cancer Registry recorded all incident tumors from 1958 with more than 90% coverage [8, 9]. The regional registries follow the same rules of registration and carry out coding, checking, and correction of the records. The notification of cancer was based on the 7th version of the International Classification of Disease (ICD-7). The diagnosis of second primary cancer is reliable in the Swedish Cancer Registry as all cancer cases included in this register were reported by both the clinician and the pathology laboratory after verification of morphological examinations. An ad hoc study showed 98% diagnostic accuracy of second neoplasms in the registry and none were found to be a metastastic cancer [10]. The Swedish Multi-Generation register recorded all the offspring born after 1931 with their biological parents. The linkage of the two registers enabled the identification of any cancer (first primary, second primary, et al) in relatives. The individuals were further linked to Total Population Register to retrieve information on socioeconomic status and place of residence.

### Family history

**Fig. 1:**
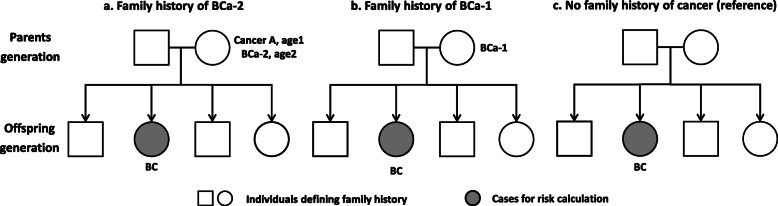
Display of how to analyze familial risk of BC using a pedigree as an example. Parents and siblings were used to define family history. Familial risk was estimated among the offspring generation. In Fig. 1a (family history of BCa-2), the mother was first diagnosed with cancer A (first primary cancer) at age1 and then BCa-2 at age2. In Fig. 1b (family history of BCa-1), the mother was diagnosed with BCa-1. In Fig. 1c (no family history of cancer), no first-degree relatives were diagnosed with any cancer. BC, breast cancer, cancer A, any cancer other than breast cancer, BCa-1, breast cancer as a first primary malignancy, BCa-2, breast cancer as a second primary malignancy

The design of the familial risk analysis is shown in Fig. 1 with an example. Family history of BCa-2 was defined as BC diagnosis after first primary cancer other than BC in FDR (parents or siblings); as shown in the example of Fig. 1a, the mother was diagnosed with BC at age2 after other cancer. Family history of BCa-1 was defined as single BC diagnosis as first primary cancer in FDR (Fig. 1b). Women without cancer diagnosis in FDR were used as reference group (Fig. 1c). The family history in the study period was independent of the time of diagnosis of BCa-1 or BCa-2 in FDRs i.e., even if the FDR was diagnosed with BCa-1 or BCa-2 after the death of an individual, this individual was considered to have a family history of BCa-1 or BCa-2, respectively.

### Study population and follow-up

The selection of the population is shown in Supplementary Fig. 1. Initially, 4,048,571 women in the offspring generation with a maximum age of 83 years were included from the Swedish population register. We removed 21,793 women with any FDR affected by multiple primary BC (BC as both first and second primary cancers), as family history of which has been used as an indicator of increased BC risk for many years [11]. It has been shown that increased BC risk was associated with the diagnosis of cancer other than BC in relatives and the number of the affected relatives had a dose-dependent effect on the risk [12]. Therefore, we further removed 1,147,108 women with any FDR affected by any other first primary cancer except those with FDR affected by BCa-2. A small number of women with an FDR affected by BCa-1 and another FDR affected by BCa-2 were removed (*N* = 395). We additionally removed women who had more than one FDR affected by BCa-1 or BCa-2, or women who had FDR affected by higher order primary cancers (such as the third or fourth primary BC) to keep the analyses comparable among different categories. We excluded the latter group, as it is difficult to distinguish which primary cancer contributed to risk modification. Ultimately, a total of 5315 women with a family history of BCa-2 were retained for the main analysis together with 2,743,777 women who had relatives without any cancer, and 115,048 women with a family history of BCa-1. We also analyzed the risk keeping families with multiple affected FDRs or FDRs with higher order primary cancers as a supplementary result (Supplementary Fig. 1). All the women were followed for a first primary invasive BC diagnosis from 1958, the year of birth, or immigration, whichever came the latest. The follow-up was ended in 2015, the year of cancer diagnosis, death or migration which came the earliest.

### Statistical analysis

The incidence rate ratio estimated from Poisson regression was used to describe relative risk (RR) by using women without FDR affected by cancer as reference group. In the model, age groups (5 years), periods (5 years), parity (number of live birth: 0, 1, 2, 3, over 3), socioeconomic status (blue-collar worker, white-collar worker, farmer, private business, professional, or other/unspecified) and place of residence (big cities, northern Sweden, southern Sweden and unspecific) were also adjusted for. Number of childbirth was considered as null parity is a risk factor for breast cancer [13]. The two-sided 95% confidence interval (CI) of the relative risk was calculated. The risk was further stratified based on the proband type (family history from mother, sister, and father and brother), age at the BC diagnosis in FDR (≤ 50 and > 50 years old), years between first primary cancer and BCa-2 diagnosis in FDR (groups based on quartiles), and sites of first primary cancer before BCa-2 in FDR. Additionally, smoking-related cancers including oral, esophageal, stomach, pancreas, lung, cervix, kidney, and bladder cancers [14], and hematological cancers including non-Hodgkin’s lymphoma, Hodgkin’s lymphoma, myeloma, and leukemia were also considered respectively as first primary cancer before BCa-2. The cumulative incidence from birth to a specific age was estimated with consideration of competing risk of being diagnosed with other cancer and death. All the statistical analyses were completed in SAS 9.4.

## Results

The median (interquartile range, IQR) age at diagnosis of the BCa-1 and BCa-2 in relatives were 58 (49–67) years and 68 (59–76) years, respectively. For women with family history of BCa-2, the median time between the first primary cancer and BCa-2 diagnoses in FDR was 8 (3-18) years. At the end of the follow-up, 5345 women, who had a FDR affected by BCa-1, were diagnosed with BC at the median age of 54 (47–62) years, and 298 women, who had a FDR affected by BCa-2, were diagnosed with BC at the median age of 54 (48–62) years.

**Fig. 2:**
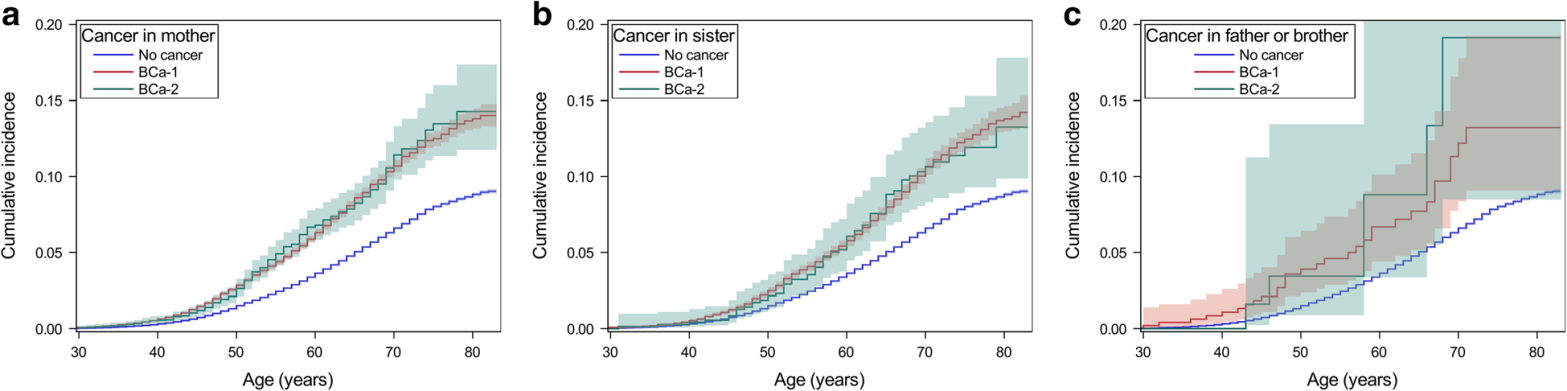
Cumulative incidence of breast cancer from birth to a specific age in women who had mother (**a**), sister (**b**) and father or brother (**c**) affected by BCa-1, BCa-2 or no cancer. BCa-1, breast cancer as a first primary malignancy, BCa-2, breast cancer as a second primary malignancy. The shading band is the 95% CI confidence interval of the cumulative incidence

Table 1 shows that the overall RR of BC was similar for women with family history of either BCa-1 (RR, 1.68, 95% CI, 1.63 to 1.73) or BCa-2 (1.68, 1.49 to1.88). When stratifying the analysis based on the age at diagnosis of BC, risk was relatively higher for women whose FDR was affected at younger age (≤50 years). The effect of age at onset was stronger for BCa-1 (difference in RR, 1.89–1.63 = 0.26) in comparison to BCa-2 (1.72–1.67 = 0.05). When BCa-2 was diagnosed in mother or sister, the risk was still equivalent to that with family history of BCa-1, but it was high for women with male FDR affected by BCa-2 (2.19, 0.98 to 4.87). However, only six BC patients had a family history of male BCa-2. The cumulative incidence of BC from birth to a specific age is displayed in Fig. 2. By age 70, the cumulative incidence was 10.7 (95%CI, 10.3 to 11.1) % and 11.4 (9.8–13.3) % for women whose mother had BCa-1 and BCa-2, respectively. For those with an affected sister, the corresponding incidences were 10.7 (10.1 to 11.2) % and 10.6 (8.3 to 13.6) %, and for those with an affected father or brother, the number was 12.2 (8.4 to 17.8) % and 19.1 (8.5 to 43.2) %. The incidence curves had overlaps between family history of BCa-1 and BCa-2 for the three type of family relationship.

**Table 1:** Breast cancer risk in women when one FDR was diagnosed with BCa-1 or BCa-2

Category	One FDR affected by BCa-1			One FDR affected by BCa-2		
	N ^a^	RR ^b^	95%CI	N ^a^	RR ^b^	95%CI
Overall	5345	**1.68**	1.63–1.73	298	**1.68**	1.49–1.88
Age at diagnosis of BC in FDR						
≤ 50 years old	967	**1.89**	1.78–2.02	11	1.72	0.95–3.10
> 50 years old	4378	**1.63**	1.58–1.68	287	**1.67**	1.49–1.88
Type of the affected FDR						
Only mother	3716	**1.70**	1.64–1.76	224	**1.69**	1.48–1.93
Only sister	1597	**1.61**	1.53–1.69	68	**1.59**	1.25–2.83
Only brother or father	32	**1.77**	1.25–2.51	6	2.19	0.98–4.87

^a^N, number of BC cases diagnosed during the follow-up in women

^b^RR was estimated from Poisson regression using individuals without cancer family history as the reference. The covariates adjusted in the model included age groups (5 years), periods (5 years), parity (number of live birth: 0, 1, 2, 3, over 3), socioeconomic status (blue-collar worker, white-collar worker, farmer, private business, professional, or other/unspecified) and place of residence (big cities, northern Sweden, southern Sweden and unspecific). Significant RRs are in bold

*BC* breast cancer, *BCa-1* breast cancer as a first primary malignancy, *BCa-2* breast cancer as a second primary malignancy, *FDR* first-degree relative, *RR* relative risk, *95%CI* 95% confidence interval

**Fig. 3:**
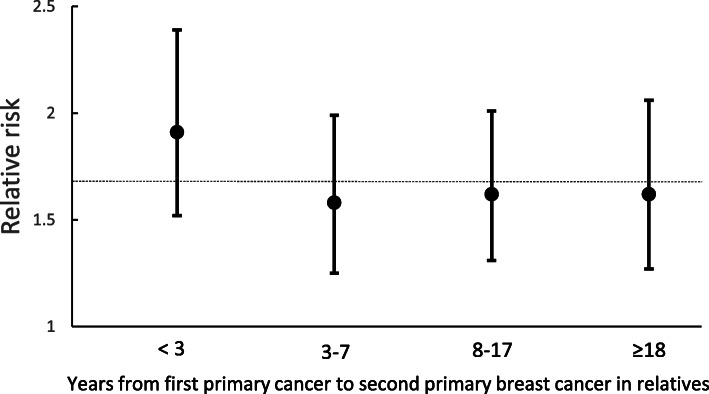
Breast cancer risk in women stratified by the time (in years) between first primary cancer and BCa-2 in FDR. The dashed line is the overall risk of BC when one FDR was affected by BCa-2. BCa-2, breast cancer as a second primary malignancy, FDR, first-degree relative

Women with a family history of BCa-2 were classified into four groups based on quartiles of the interval (3, 8 and 18 years) between the first primary cancer and BCa-2 diagnosed in relatives (Fig. 3). When relatives were diagnosed with BCa-2 soon after the first primary cancer (within 3 years), the risk of BC was 1.91 (1.52 to 2.39), and it remained stable for BCa-2 that occurred after 3 years.

BC risk with a family history of BCa-2 was further estimated according to the sites of the first primary cancer diagnosed before BCa-2 in FDR (Table 2). The risk varied for different first primary cancers, among which, cancers of stomach, colorectal, endometrium, ovary, nervous system and endocrine gland, and non-Hodgkin lymphoma as well as smoking-related and hematological cancers had a significantly increased RR. Specifically, for the tumor in the nervous system, two-thirds of the relatives were diagnosed with meningioma. Among the 20 relatives with endocrine gland tumors, 18 had parathyroid cancers.

**Table 2:** Breast cancer risk in women stratified by site of first primary cancer diagnosed before BCa-2 in their FDRs

Site of first primary cancer before BCa-2 in FDR	N ^a^	RR ^b^	95%CI	
UAT ^c^	5	1.21	0.50	2.90
Stomach ^c^	6	**3.92**	1.76	8.72
Small intestine	2	1.61	0.40	6.42
CRC	44	**1.60**	1.19	2.15
Liver	3	2.10	0.68	6.52
Lung ^c^	5	1.00	0.42	2.40
Cervix ^c^	16	1.46	0.89	2.38
Endometrium	60	**2.07**	1.60	2.67
Ovary	24	**2.82**	1.89	4.21
Female genital	2	0.79	0.20	3.17
Prostate	3	2.58	0.83	7.99
Kidney ^c^	10	1.44	0.77	2.68
Bladder ^c^	8	1.00	0.50	2.00
Melanoma	21	1.33	0.87	2.04
Skin	11	1.11	0.61	2.01
Nervous system	18	**1.94**	1.22	3.07
Thyroid	10	1.54	0.83	2.86
Endocrine gland	20	**2.05**	1.32	3.18
Bone	1	0.80	0.11	5.69
Connective tissue	4	1.89	0.71	5.05
NHL ^d^	12	**2.84**	1.61	5.00
Hodgkin lymphoma ^d^	3	2.06	0.67	6.40
Myeloma ^d^	1	1.59	0.22	11.29
Leukemia ^d^	4	0.85	0.32	2.26
CUP	5	2.24	0.93	5.38
Smoking related cancers	50	**1.34**	1.02	1.77
Hematological cancers	20	**1.82**	1.17	2.81
Any cancer other than BC	298	**1.68**	1.49	1.88

^a^N, number of BC cases diagnosed during the follow-up in women

^b^RR was estimated from Poisson regression using individuals without cancer family history as the reference. The covariates adjusted in the model included age groups (5 years), periods (5 years), parity (number of live birth: 0, 1, 2, 3, over 3), socioeconomic status (blue-collar worker, white-collar worker, farmer, private business, professional, or other/unspecified) and place of residence (big cities, northern Sweden, southern Sweden and unspecific). Significant RRs are in bold

^c^Cancers that were included as smoking-related cancers

^d^Cancers that were included as hematological cancers

*BC* breast cancer, *BCa-2* breast cancer as a second primary malignancy, *FDR* first-degree relative, *UAT* upper aerodigestive tract, *CRC* colorectal cancer, *NHL* non-Hodgkin lymphoma, *CUP* cancer of unknown primary, *RR* relative risk, *95%CI* 95% confidence interval

We present the results without removing women with more than one FDR affected by BCa-1 or BCa-2 and women with FDRs affected by cancer after BCa-1 and BCa-2 in Supplementary Table 1. The BC risks for family history of BCa-1 (1.74, 1.69 to 1.78) and BCa-2 (1.77, 1.60 to 1.96) both increased but were still similar. In the analysis considering the number of the affected relatives, high risk was associated with more than one FDR diagnosed with BCa-2 (4.33, 1.94 to 9.63), but the case number was small (*N* = 2).

## Discussion

With the increasing number of the BCa-2, it is of great importance to investigate if familial risk differs between a family history of BCa-1 and BCa-2. Most of the studies involved with a family history of multiple primary cancers were set up in families with hereditary cancer syndrome [15]. No study has systematically investigated the BC risk when FDRs have BCa-2 following a non-breast cancer diagnosis. The principal finding of this nationwide population-based cohort study was that a diagnosis of either BCa-1 or BCa-2 is associated with a similar BC risk for their family members,

We excluded women who had multiple FDRs affected by BCa-2 and women who had FDRs affected by BCa-2 then later developed with third, fourth or even fifth primary cancers, which could be the reason that we observed slightly lower familial risk (1.68). After removing those restrictions, the familial risk was comparable to those reported from other studies [2–4]. The risk pattern for proband type did not differ in the family history of BCa-1 and BCa-2. Consistent with other studies, the familial risk was higher when the relatives diagnosed with BC at a younger age. It has been reported that early-onset cancers are more likely to have a hereditary background than late-onset ones [16]. In a recent study, negative association was found between age of onset of BC and the fraction of BC-associated germline mutation [17]. Interestingly, we did not observe an apparent difference in risk between familial early- and late-onset BCa-2. We speculate that this was due to the late-onset for most of the BCa-2 compared to BCa-1 and the current sample size was not able to provide enough power to distinguish the familial risk associated with the early- and late-onset BCa-2 in FDRs.

The development of the second primary cancers is linked to various factors which is partially indicated by the interval between first and second primary cancer diagnoses. The intensified medical surveillance increases the chance of second primary cancers diagnosed right after the first primary and those cancers are unlikely to be caused by the treatment from the first primary. Instead, treatment-related second primary cancers develop after several years from the first primary cancer diagnosis. In the analysis stratified by the time between the first primary cancer and BCa-2 in relatives, despite fluctuation, the familial risks were largely consistent, implying the influences of medical surveillance and cancer treatments on familial risk of BC is limited.

The finding of the varied risk for a family history of BCa-2 after some specific cancers, indicates that the site of cancer before BCa-2 gives a better prediction of BC risk. For endometrial and ovarian cancers, the increased risk can be explained by shared reproductive factors, the germline mutation manifested in hereditary breast and ovarian cancer syndrome or the interaction between them. The elevated risk with a FDR affected by first primary stomach cancer may be related to a type of hereditary diffuse gastric cancer associated with germline mutation of *CHD1* [18]. For meningiomas, hormone level may have played role in the elevated BC risk in daughters [19]. As for endocrine tumors, the increased risk of BC may be associated with multiple endocrine neoplasia type 1 syndromes [20]. For smoking-related cancers as the first primary, with the exception of stomach cancer, a relatively lower risk of BC was found in FDR with BCa-2 compared to that with BCa-1. The further development of BCa-2 in lung cancer patients could be due to radiotherapy that could be hardly shared by the family members. No study has reported radiotherapy-induced BC in lung cancer, possibly due to the poor survival of lung cancer, but a dose-response relationship for BC risk has been demonstrated in numerous medical radiation studies and researches on other malignancies such as Hodgkin lymphoma [21]. However, lung cancer only accounted for one-tenth of the smoking-related cancers, so other factors may also have contributed to the relatively lower risk. Smoking is also a risk factor for BC despite small effect size [22]. BC may have developed after smoking-related cancer due to smoking instead of strong hereditary factors. This is also supported by the late onset of BC (median age 60, IQR, 51–67) in daughters of relatives with first primary smoking-related cancers compared to the overall age of onset of the 298 BC patients (54, 48–62).

### Clinical implication

Family history has been an important determinant for the assessment of BC risk in commonly used models including NCI-Gail, BCSC, IBIS and BOADICEA [23, 24]. The dimension of the family history contains the degree and number of affected family members, diagnostic age of BC, bilateral or ipsilateral BC in relatives. None of them considered the order of the primary BC (either BCa-1 or BCa-2 here) diagnosed in FDRs. The comparable BC risk for family history of BCa-1 and BCa-2 in the current study suggests that a diagnosis of BCa-2 in FDR can be treated as the same as BCa-1 regarding BC risk prediction. However, the varied familial risk according to the cancer site before BCa-2 implies the necessity to include the first primary cancer into the consideration for family history of BCa-2. For example, BC risk is high for women with FDRs affected by a BCa-2 after first primary stomach, endometrial and ovarian cancers and non-Hodgkin lymphoma. Although, due to the relatively small sample size, further investigation is warranted.

### Strengths and limitations of the study

By using the Swedish national registries, we were able to obtain accurate family relationships and order of multiple primary cancers without information bias. We implemented very strict analyses to control the possible influences (such as the number of relatives with cancers) on the risk estimation. Analyses without the restrictions were also performed to generalize the results. We conducted multiple stratification analyses to characterize BC risk with the family history of BCa-2. The family history can be defined in dynamic and static scale depending on if the presence of the disease in FDRs is considered as time-varying and time-fixed. The two methods generate a similar estimation of relative risk [25, 26], so the results from the current study using the static method would not deviate much from that using the dynamic method. Some limitations need to be addressed. The case number was not adequate to estimate risk for family history from male relatives, and the same issue persisted with some specific first primary cancers. In addition to parents and siblings, offspring is usually defined as an FDR, but we did not include it in the analysis, as it is very rare for parents to have BC after BCa-2 diagnosis in offspring since the median diagnostic age of BCa-2 is 68. Our study did not adjust for the number of sisters although sibling risk of BC has been reported to increase with the increasing number of sisters [27]. Data on some BC risk factors like smoking, physical activity and diet were unavailable along with information on treatment for the first primary cancer and individual germline mutation, making some of the discussion on the association with other first primary cancers speculative at best. The hormonal status of BC was not known, which could have given more precise risk estimation. We did not find any explanation for high risk associated with diagnosis of non-Hodgkin lymphoma as first primary cancer in FDR and more studies are needed to investigate the association.

## Conclusions

As far as we are aware, we for the first time, provided information on BC risk assessment for women with family history of BCa-2. The similar BC risk indicates that women with FDR affected by BCa-2 can be managed similarly as those with FDR affected by BCa-1, regarding BC prevention and screening. In addition, for those with family history of BCa-2, the familial risk varies most according to the site of first primary cancer in FDR, which should be considered in the clinical practice.

## Supplementary Information

**Additional file 1.** **Supplementary Figure 1.** The flowchart of the population selection. **Supplementary Table 1.** Breast cancer risk in women when FDRs were diagnosed with BCa-1 or BCa-2.*

## Data Availability

The data that support the findings of this study are available from Lund University but restrictions apply to the availability of these data, which were used under license for the current study and so are not publicly available. Any request regarding the data from this study should go to the last author (J.J.).
