# Supplementary material for: Family history of breast cancer as a second primary malignancy in relatives: a nationwide cohort study
Source: BMC Cancer. 2021 Nov 12;21:1210. doi: 10.1186/s12885-021-08925-y (PMC8590230; doi:10.1186/s12885-021-08925-y)
Supplement: Supplementary file 1 — Additional file 1. Supplementary Figure 1. The flowchart of the population selection. Supplementary Table 1. Breast cancer risk in women when FDRs were diagnosed with BCa-1 or BCa-2.* [file 12885_2021_8925_MOESM1_ESM.docx]

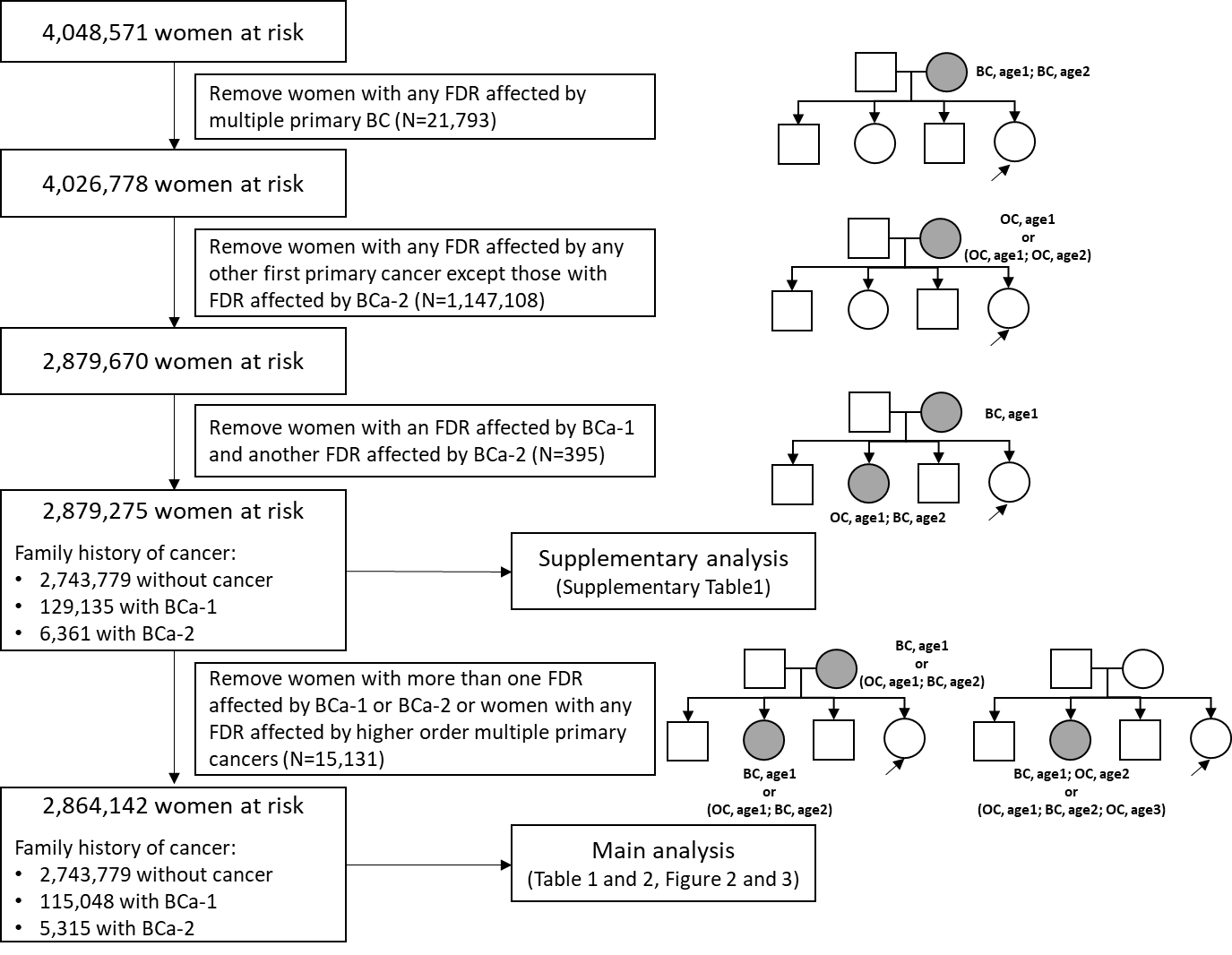


Supplementary Figure 1. The flowchart of the population selection. The pedigrees on the right display the individuals that were removed in each step with corresponding family history. The individual with arrow is the one for risk estimation. If one individual was diagnosed with two cancers, age1 was always less than age2. For those with three cancer, age1 was less than age2 and age2 less than age3. BC, breast cancer, BCa-1, breast cancer as a first primary malignancy, BCa-2, breast cancer as a second primary malignancy, FDR, first-degree relative, OC, other cancer

Supplementary Table 1. Breast cancer risk in women when FDRs were diagnosed with BCa-1 or BCa-2.*

| Category | FDRs affected by BCa-1 | | | FDRs affected by BCa-2 | | |
| --- | --- | --- | --- | --- | --- | --- |
|  | N ^a^ | RR ^b^ | 95%CI | N ^a^ | RR ^b^ | 95%CI |
| Overall | 6496 | **1.74** | 1.69-1.78 | 377 | **1.77** | 1.60-1.96 |
| No. of affected FDRs |  |  |  |  |  |  |
| one FDR | 6055 | **1.70** | 1.65-1.74 | 375 | **1.77** | 1.60-1.96 |
| > one FDR | 441 | **2.60** | 2.37-2.86 | 2 | 2.84 | 0.71-11.4 |

*The study population in this table was the extension of the study population in the main analysis. In the main analysis, women with family history of BC were only those with one FDR affected by BCa-1 or BCa-2 and those with no other cancer diagnosed after BCa-1 or BCa-2. Women with family history of BCa-1 in this table can have more than one FDR affected by BCa-1 or can have FDRs affected by other cancer after BCa-1. Similarly, women with family history of BCa-2 can have more than one FDR affected by BCa-2 or can have FDRs affected by other cancer after BCa-2.

^a^, N, number of BC cases diagnosed during the follow-up in women.

^b^, RR was estimated from Poisson regression using women without cancer family history as the reference. The covariates adjusted in the model included age groups (5 years), periods (5 years), parity (number of live birth: 0, 1, 2, 3, over 3), socioeconomic status (blue-collar worker, white-collar worker, farmer, private business, professional, or other/unspecified) and place of residence (big cities, northern Sweden, southern Sweden and unspecific). Significant RRs are in bold.

BCa-1, breast cancer as first primary malignancy, BCa-2, breast cancer as second primary malignancy, FDR, first-degree relative, RR, relative risk, 95%CI, 95% confidence interval
